# The recently introduced *Aedes albopictus* in Tunisia has the potential to transmit chikungunya, dengue and Zika viruses

**DOI:** 10.1371/journal.pntd.0008475

**Published:** 2020-10-02

**Authors:** Chloé Bohers, Laurence Mousson, Yoann Madec, Marie Vazeille, Adel Rhim, Youmna M’ghirbi, Ali Bouattour, Anna-Bella Failloux

**Affiliations:** 1 Institut Pasteur, Department of Virology, Arboviruses and Insect Vectors, Paris, France; 2 Institut Pasteur, Department of Global Health, Epidemiology of Emerging Diseases, Paris, France; 3 Laboratoire Virus, Vecteurs et Hôtes, Institut Pasteur de Tunis, Université de Tunis El Manar, Tunis-Belvédère, Tunisia; Centers for Disease Control and Prevention, UNITED STATES

## Abstract

The mosquito *Aedes albopictus* was detected for the first time in Tunisia in 2018. With its establishment in the capital city of Tunis, local health authorities fear the introduction of new human arboviral diseases, like what happened in Europe with unexpected local cases of chikungunya, dengue and Zika. Even though this mosquito is competent to transmit the arboviruses mentioned above, the transmission level will vary depending on the couple, mosquito population and virus genotype. Here, we assessed the vector competence of *Ae*. *albopictus* Tunisia by experimental infections with chikungunya (CHIKV), dengue (DENV), and Zika (ZIKV) viruses. We found that *Ae*. *albopictus* Tunisia was highly competent for CHIKV (transmission efficiency of 25% at 21 post-infection) and to a lesser extent, for ZIKV (8.7%) and DENV (8.3%). Virus was detected in mosquito saliva at day 3 (CHIKV), day 10 (ZIKV) and day 21 (DENV) post-infection. These results suggest that the risk of emergence of chikungunya is the highest imposing a more sustained surveillance to limit *Ae*. *albopictus* populations in densely populated urban dwellings and at the entry points of travelers returning from CHIKV-endemic regions.

## Author summary

After Algeria and Morocco, the mosquito *Aedes albopictus* was detected in 2018 in Tunisia. This invasive species has continued its global expansion and then, extended the fear of introducing *Aedes*-transmitted arboviral diseases in countries where people are immunologically naïve. We demonstrated, using experimental infections, that *Ae*. *albopictus* mosquitoes from Tunisia were more competent to chikungunya virus than to Zika virus and dengue virus. These results are critical to design a control strategy targeting *Ae*. *albopictus* populations in regions hosting travelers returning from arboviral-endemic regions.

## Introduction

Ten mosquito species belonging to the *Aedes* (Stegomyia) genus (Diptera:Culicidae) are reported in Tunisia [1, 2]: *Ae*. *berlandi*, *Ae*. *caspius*, *Ae*. *detritus*, *Ae*. *echinus*, *Ae*. *geniculatus*, *Ae*. *mariae*, *Ae*. *pulcritarsis*, *Ae*. *vexans*, *Ae*. *vittatus*, and lastly, *Ae*. *albopictus*. This last species was detected for the first time in Tunisia in 2018 [3]. It is considered one of the most successful invasive species during these past few decades, having spread to several countries on all continents except Antarctica [4]. In the Mediterranean region, *Ae*. *albopictus* was first detected in Albania in 1979 [5] and again, in Italy in 1990 [6]; it became quickly well established in all European Mediterranean countries [7]. In North Africa, it was reported in Algeria in 2014–15 [8, 9] and in Morocco in 2015 [10].

This species was responsible for serious outbreaks of arboviral diseases since it is a competent vector for at least 26 arboviruses [11, 12]. In Europe, the species has been involved in local transmission of chikungunya virus (CHIKV) [13], dengue virus (DENV) [14], and more recently, Zika virus (ZIKV) [15]. Among all arboviruses transmitted by mosquitoes, DENV (*Flavivirus*, *Flaviviridae*) causes the highest incidence in terms of human morbidity and mortality, with 300 million people infected each year resulting in 22,000 deaths [16]. Historically, four serotypes (DENV-1, -2, -3, and -4) have been described within the DENV antigenic complex [17]. Besides, CHIKV (*Alphavirus*, *Togaviridae*) is an arthritogenic virus composed of three major genotypic lineages: Asian, East/Central/South African (ECSA), and West African [18]. CHIKV was responsible for a large-scale epidemic within the Indian Ocean region in 2004–5 [19] where the principal vector was *Ae*. *albopictus*, instead of the more traditional *Aedes aegypti* [20]. The shift in vector species was associated with a single alanine to valine substitution at position 226 of the CHIKV E1 glycoprotein [21, 22]. The ECSA strain was responsible for millions cases in Asia, Africa, and Europe [23]. Lastly, ZIKV (*Flavivirus*, *Flaviviridae*) hit recently billions of immunologically naïve people in regions where ZIKV-competent mosquitoes predominate. After the South Pacific region [24, 25], ZIKV reached the American continent in 2015 [26] where ZIKV of the Asian clade circulated [27, 28]. The mosquito *Ae*. *aegypti* seems to be the main vector of ZIKV and *Ae*. *albopictus*, a secondary vector [29, 30].

The three arboviruses are transmitted by the two anthropophilic mosquitoes *Ae*. *aegypti* and *Ae*. *albopictus*. They are typical human-adapted arboviruses having lost the need for an enzootic cycle to trigger outbreaks [31]. Socio-economic changes affecting Tunisia these last decades have favored the rapid development of urban centers where conditions are met to favor the proliferation of domestic mosquitoes, multiple standing water as larval breeding sites and dense human populations as source for mosquito blood feeding [2]. Following the detection of *Ae*. *albopictus* in Algeria and Morocco in 2015, the Ministry of Health of Tunisia implemented a country wide mosquito survey from April 2015 to September 2018 in ports and airports [3]. *Aedes albopictus* was detected in October 2018 in Carthage (20 km apart from the capital city of Tunis) facing the Italian islands of Pantelleria and Lampedusa where it was first identified in 2015 [32]. In Tunisia, the most prevalent mosquito-borne virus is West Nile virus (*Flavivirus*, *Flaviviridae*) with outbreaks reported in 1997 [33], 2003 [34], 2012 [35]. Occasionally, Sandfly fever viruses (*Phlebovirus*, *Bunyaviridae*) were detected [36] as well as Usutu virus (*Flavivirus*, *Flaviviridae*) [37]. Therefore, *Ae*. *albopictus* may introduce a new chain of local transmission of DENV, CHIKV and ZIKV fostered by a high frequency of viremic travelers, high densities of competent vectors, and suitable environmental conditions. Tunisia is an open touristic country receiving each year 8 millions of tourists from all over the world and local population is immunologically naïve to the three arboviruses. Higher densities of *Ae*. *albopictus* in urban settings would be expected since the number of infested houses has increased in 2019 during the warm season (Bouattour A., com. pers) and as it is the case today in Algeria [38]. Thereby it seems critical to evaluate the susceptibility of a local *Ae*. *albopictus* population to major arboviruses. Here we assessed the vector competence of *Ae*. *albopictus* Tunisia to CHIKV, DENV, and ZIKV using experimental infections to describe viral infection, dissemination and transmission.

## Materials and methods

### Ethic statements

Animals were housed in the Institut Pasteur animal facilities (Paris) accredited by the French Ministry of Agriculture for performing experiments on live rodents. Work on animals was performed in compliance with French and European regulations on care and protection of laboratory animals (EC Directive 2010/63, French Law 2013–118, February 6th, 2013). All experiments were approved by the Ethics Committee #89 and registered under the reference APAFIS#6573-201606l412077987 v2.

### Mosquito collections

*Aedes albopictus* Tunisia mosquitoes (76 adults,70 females and 6 males) were collected in October 2018 in Carthage, Amilcar, and La Marsa, 20 km from the capital city of Tunis [3] and the F3 generation was used for experimental infections. A laboratory colony, *Ae*. *albopictus* Providence originally collected in 2010 in La Providence on La Réunion Island and maintained since then in insectaries, was used as control; this population was involved in major outbreaks of CHIKV [19] and DENV [39]. Mosquitoes were reared in standardized conditions. After egg hatching, 200 larvae were distributed in pans containing 1 liter of dechlorinated water and a yeast tablet renewed every 2 days, and maintained at 25±1°C. Pupae were individually collected in bowls placed in cages where adults emerged. Adults were fed with a 10% sucrose solution and kept at 28±1°C with a 16L:8D cycle and 80% relative humidity.

### Viral strains

CHIKV strain (06.21) was isolated from a patient on La Réunion Island in 2005 [40]; viral stocks were produced after two passages on C6/36 cells. DENV-2 strain was isolated from a human serum collected in Bangkok (Thailand) in 1974 [41]; after 2 passages in *Ae*. *albopictus*, 2 others in *Toxorhynchites amboinensis*, and one in *Ae*. *aegypti* by intrathoracic inoculation, viral stocks were obtained by inoculating C6/36 cells. ZIKV strain (NC-2014-5132) was originally isolated from a patient in April 2014 in New Caledonia [42]; viral stocks were produced after 5 passages on Vero cells. All viral stocks were stored at -80°C until use.

### Mosquito infections

Five to six batches of 60 females (7–10 day old) were isolated in boxes and exposed to an infectious blood meal containing 1.4 mL of washed rabbit erythrocytes, 700 μL of viral suspension and ATP at 1 mM as a phagostimulant. The titer of the blood meal was at 10^7^ focus-forming unit (ffu)/mL for CHIKV and DENV, and 10^7^ TCID_50_/mL for ZIKV. The feeding procedure used a Hemotek system with a pork intestine as membrane covering the base of a feeder maintained at 37°C. After 15 min of feeding, engorged mosquitoes were transferred in cardboard containers and maintained with 10% sucrose under controlled conditions (28±1°C, relative humidity of 80%, 16L:8D cycle) for up to 21 days. *Ae*. *albopictus* Tunisia were examined at 3, 7, 10, 14, and 21 days post-infection (dpi) when exposed to CHIKV and DENV, and 7, 10, 14, and 21 to ZIKV while mosquitoes from La Réunion as control were only examined at 14 dpi based on our previous published data [22, 43, 44]. For each virus, 21–30 mosquitoes were examined at each dpi.

### Processing mosquitoes

Surviving mosquitoes at each examined dpi, were cold anesthetized on ice. Then legs and wings were removed and the proboscis was inserted into a pipette tip containing 5 μL of fetal bovine serum (FBS). After 30 min, the tip content was transferred in 45 μL of L15 medium [45]. Then, abdomen was detached from the thorax and head. These two samples were separately ground in 300 μL of Leibovitz L15 medium (Invitrogen, CA, USA) supplemented with 2% FBS, and centrifuged at 10,000×g for 5 min at +4°C. Abdomen (containing the midgut), head/thorax (if viruses are detected, it means that head/thorax contains viruses having escaped from the midgut) were tested respectively for infection and dissemination while saliva was titrated to estimate transmission.

### Vector competence indices

To measure the vector competence, three indices were evaluated at the two main steps in the path of the virus inside mosquitoes after the infectious blood meal: (i) infection rate (IR) corresponding to the proportion of mosquitoes with infected midgut among engorged mosquitoes, (ii) dissemination rate (DR) referring to the proportion of mosquitoes having succeeded in disseminating the virus inside the mosquito general cavity (namely the hemocele) among mosquitoes with infected midgut, and (iii) transmission rate (TR) which measures the final step corresponding to the proportion of mosquitoes with infectious saliva among mosquitoes having disseminated the virus. DR measures the efficiency of the midgut as a barrier to the dissemination of the virus inside the hemocele; the higher it is, the less the midgut is a brake to the dissemination of the virus. In addition to DR, TR measures the efficiency of the salivary glands as a barrier to the excretion of the virus in the saliva; as DR, the higher it is, the less the salivary glands play the role of barrier to the transmission of the virus.

Moreover, two other indices are used to describe viral dissemination (dissemination efficiency (DE), proportion of mosquitoes with disseminated infection) and transmission (transmission efficiency (TE), proportion of mosquitoes able to transmit) by considering the total number of surviving mosquitoes; DE and TE have the advantage of giving a rapid estimate without measuring the effectiveness of the previous steps (infection for DE and dissemination for TE).

### Viral titration

CHIKV and DENV were titrated by focus fluorescent assay (ffu). Because ZIKV cannot produce distinct viral foci on mosquito cells, it was titrated by plaque forming assay (pfu).

### Focus forming assay on C6/36 cells

For mosquitoes exposed to CHIKV and DENV, homogenates and saliva were titrated by focus fluorescent assay on *Ae*. *albopictus* C6/36 cells [46]. After serial dilutions, samples were inoculated onto C6/36 cells in 96-well plates. After a 3-day incubation period for CHIKV, and 5-day for DENV at 28°C, cells were stained using hyper-immune ascetic fluid specific to each virus as the primary antibody: provided by the French National Reference Center for Arbovirus at the Institut Pasteur for CHIKV and Ms X Dengue complex MAB 8705 (Millipore, MA, USA) for DENV. Alexa Fluor 488 goat anti-mouse IgG (Life Technologies, CA, USA) was used as the secondary antibody.

### Plaque forming assay on Vero cells

For ZIKV, homogenates were serially diluted and inoculated onto monolayers of Vero cells in 96-well plates. After a 7-day incubation period at 37°C, cells were stained with a solution of safranine (0.5% in 10% formaldehyde and 10% ethanol). Presence of viral particles was assessed by CPE detection. Saliva was titrated on monolayers of Vero cells in 6-well plates incubated 7 days under an agarose overlay.

### Statistical analysis

The effect of virus on IR, DR and TR was investigated using logistic regression models, as were investigated the effects of dpi and of viral load. Viral loads were compared between groups using Mann-Whitney non-parametric test. Then, viral load measured in abdomen that best discriminated i) mosquitoes that disseminated from those that did not and ii) mosquitoes that transmitted from those that did not, was investigated using ROC (Receiver Operating Characteristic) curves which are graphical plots that represent the ability of a continuous marker to correctly classify a binary outcome. Viral load measured in head/thorax that best discriminated mosquitoes that transmitted from those that did not, was also investigated using ROC curves. Based on the ROC curves, thresholds that best discriminated mosquitoes with and without dissemination on the one hand, and with and without transmission in the other hand were identified. Statistical analyses were conducted using the Stata software (StataCorp LP, Texas, USA). p-values < 0.05 were considered significant.

## Results

### *Aedes albopictus* mosquitoes from Tunisia better disseminate and transmit CHIKV

A total of 357 mosquitoes were exposed to an infectious blood meal and 54.6% (195/357) were abdomen-infected: 81.3% (109/134) for CHIKV, 26.2% (37/141) for DENV and 59.8% (49/82) for ZIKV. The logistic regression model also showed the significantly reduced IR with ZIKV and even further with DENV (Table 1; P < 0.001). Out of the 195 infected mosquitoes, dissemination occurred in 139 (71.7%), with DR of 90.8% with CHIKV, 48.6% with DENV and 44.9% with ZIKV. Again, the logistic regression showed a significantly reduced DR with DENV and ZIKV (P < 0.001). Finally, out of the 139 mosquitoes with dissemination, transmission was observed in 53 (38.1%), TR being 45.4% with CHIKV, 17.6% with DENV and 22.7% with ZIKV; as compared to TR with CHIKV, it was significantly reduced with DENV and only tended to be lower with ZIKV. The univariate logistic regression showed no effect of dpi on IR, but a potential effect on DR and on TR (Table 1, P = 0.77, 0.09 and 0.08, respectively).

**Table 1:** Univariate logistic regression analyses for infection, dissemination and transmission of CHIKV, DENV and ZIKV in *Ae*. *albopictus* Tunisia.

	Crude OR (95% CI) for IR	P	Crude OR (95% CI) for DR	P	Crude OR (95% CI) for TR	P
Virus CHIKV DENV ZIKV	1 **0.08 (0.05–0.14)** **0.34 (0.18–0.63)**	<0.0001	1 **0.10 (0.04–0.24)** **0.08 (0.03–0.19)**	<0.0001	1 **0.26 (0.07–0.95)** 0.35 (0.12–1.03)	0.019
Days post-infection ≤7 [8–14] [15–21]	1 0.84 (0.52–1.36) 0.91 (0.52–1.60)	0.77	1 1.70 (0.85–3.41) 2.48 (1.03–5.97)	0.09	1 2.23 (0.98–5.10) 1.02 (0.39–2.66)	0.08

In bold, significant values (P < 0.05)

**Fig 1:**
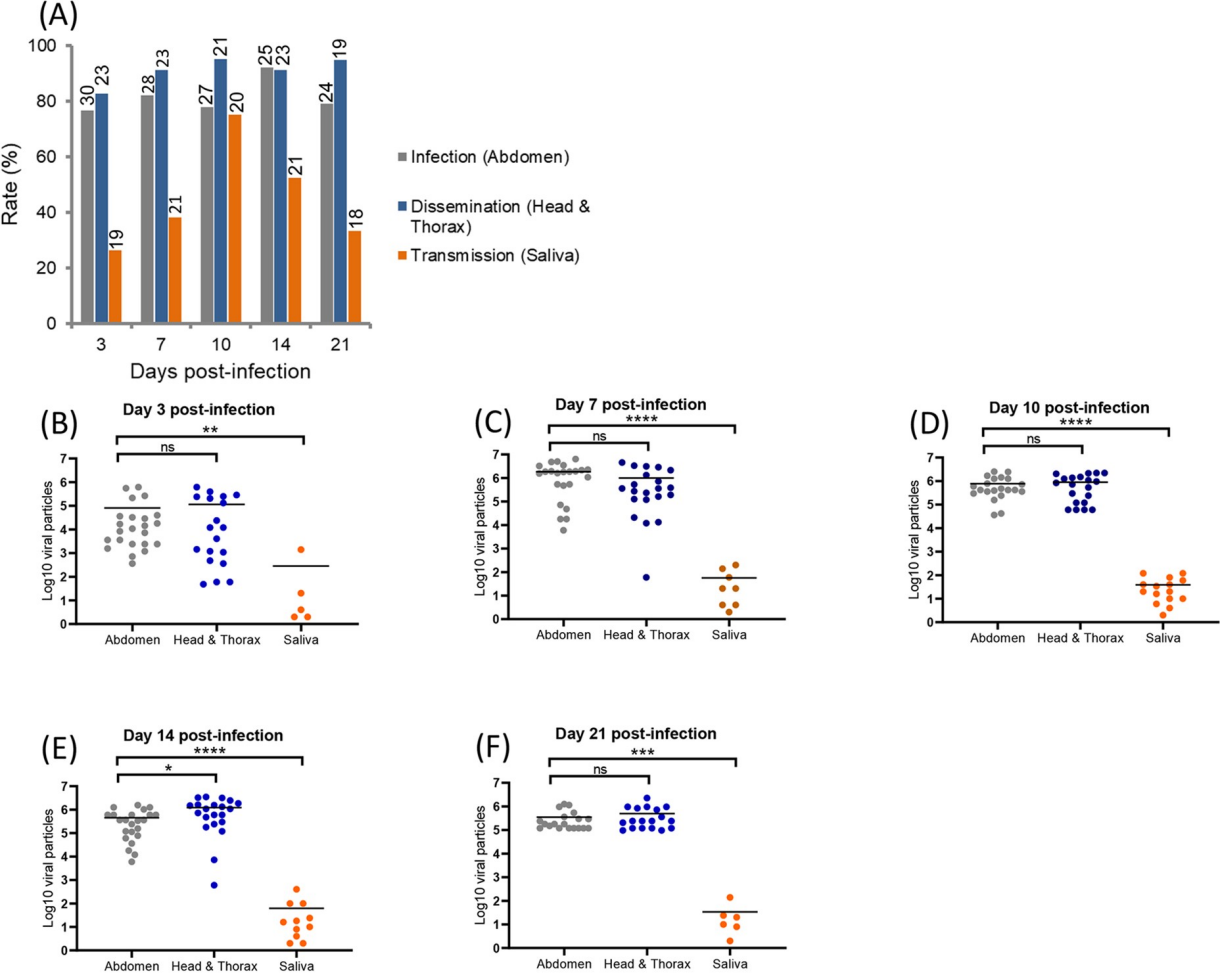
**CHIKV infection, dissemination, transmission (A) and viral loads in abdomen, head/thorax and saliva (B-F) in *Aedes albopictus* Tunisia at different days post-infection (3, 7, 10, 14 and 21).** Mosquitoes were infected with a blood meal at a titer of 10^7^ ffu/mL and at each day post-infection, mosquitoes were processed to estimate the viral load in abdomen, head/thorax and saliva by titration on cells. On top of the bars are the numbers of mosquitoes tested (A). Means are represented by horizontal bars (B-F). ns, non-significant, **p ≤ 0.01, ***p ≤ 0.001, ****p ≤ 0.0001.

With CHIKV specifically, IR ranged from 76.7% (3 dpi) to 79.2% (21 dpi) and among mosquitoes with an infected midgut, more than 80% (DR from 82.6% at 3 dpi to 94.7% at 21 dpi) were able to disseminate the virus from the midgut epithelium into the hemocele (i.e. efficient crossing of the midgut barrier) (Fig 1A). Among mosquitoes with disseminated infection, at least 26.3% (3 dpi) were able to transmit the virus expelled with mosquito saliva (i.e. efficient crossing of the salivary glands barrier); a TR of 75% were detected at 10 dpi (Fig 1A). More globally, when considering mosquitoes with disseminated infection among tested mosquitoes (with and without an infected midgut), DE reached its maximum value at 14 dpi (84%) (S1A Fig), and when examining mosquitoes with infected saliva among tested mosquitoes (with and without disseminated infection), TE reached the maximum of 55.5% at 10 dpi (S1A Fig). When estimating the number of viral particles (Fig 1B–1F), viral loads were similar in abdomen (a mean of 10^4.9^ viral particles at 3 dpi to 10^5.5^ at 21 dpi) and head/thorax (a mean of 10^5.0^ viral particles at 3 dpi to 10^5.7^ at 21 dpi) at each dpi (Mann-Whitney test: P > 0.05) whereas there were 1,000 to 20,000 times less viral particles in mosquito saliva (a mean of 10^2.4^ viral particles at 3 dpi to 10^1.5^ at 21 dpi).

**Fig 2:**
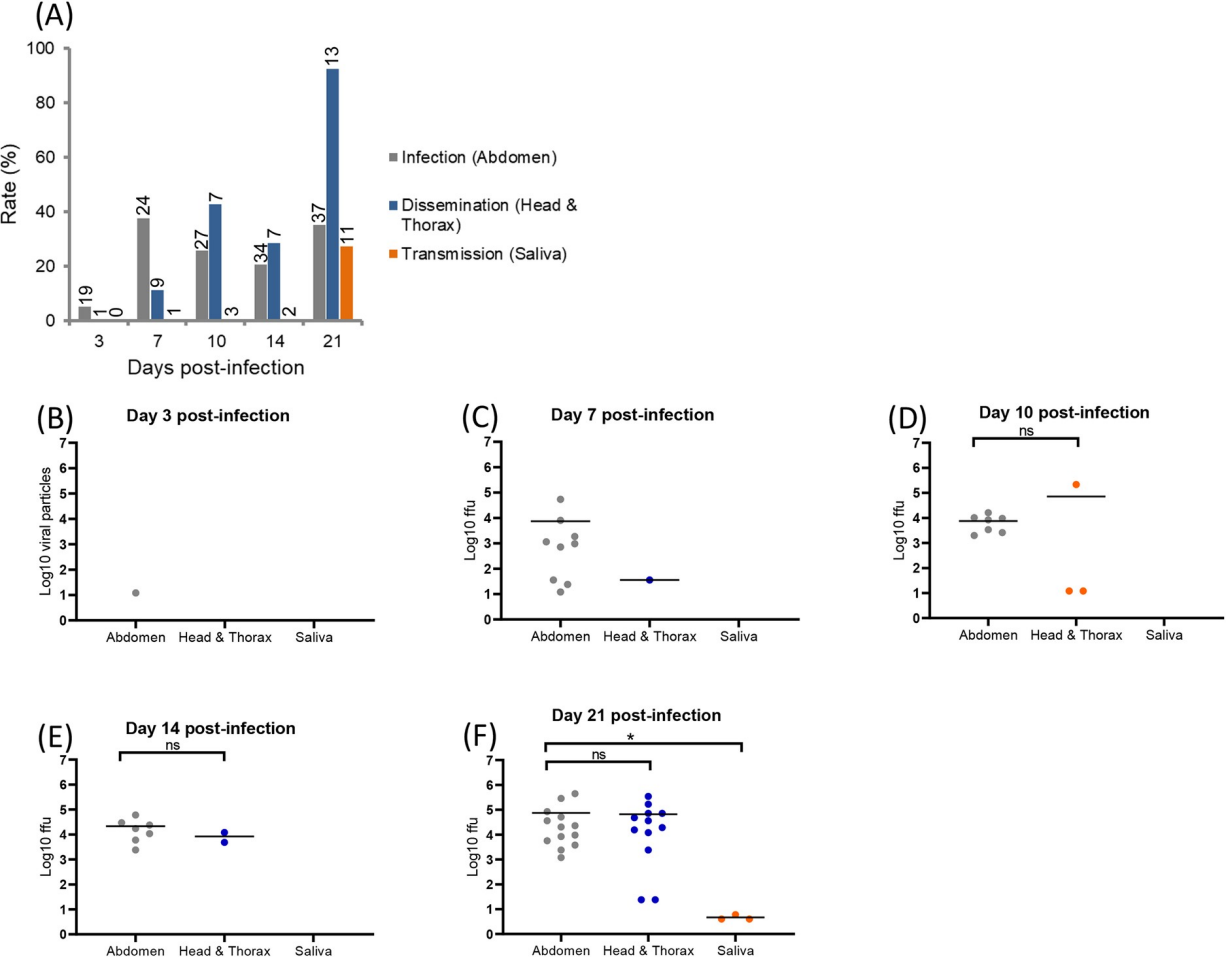
**DENV infection, dissemination, transmission (A) and viral loads in abdomen, head/thorax and saliva (B-F) in *Aedes albopictus* Tunisia at different days post-infection (3, 7, 10, 14 and 21).** Mosquitoes were infected with a blood meal at a titer of 10^7^ pfu/mL and processed as described in Fig 1. On top of the bars are the numbers of mosquitoes tested (A). Means are represented by horizontal bars (B-F). ns, non-significant, *p < 0.05.

When infected with DENV, IR ranged from 5.3% (3 dpi) to 35.1% (21 dpi), DR from 11.1% at 7 dpi to 92.3% at 21 dpi, and TR reached the value of 27.3% at 21 dpi (Fig 2A). Viral dissemination started from 7 dpi (DE of 4.2%) and transmission only at 21 dpi (TE of 8.3%) (S1B Fig). When quantifying viral particles in abdomen, head/thorax, and saliva at 3, 7, 10, 14, and 21 dpi (Fig 2B–2F), viral loads in abdomen ranged from a mean of 10^1.1^ viral particles at 3 dpi to 10^4.9^ at 21 dpi and in head/thorax, from 10^1.55^ viral particles at 7 dpi to 10^4.8^ at 21 dpi. Viral loads were similar in abdomen and head/thorax at each dpi (Mann-Whitney test: P > 0.05). Viral particles in saliva were only detected at 21 dpi with a mean of 10^0.7^ particles per saliva.

**Fig 3:**
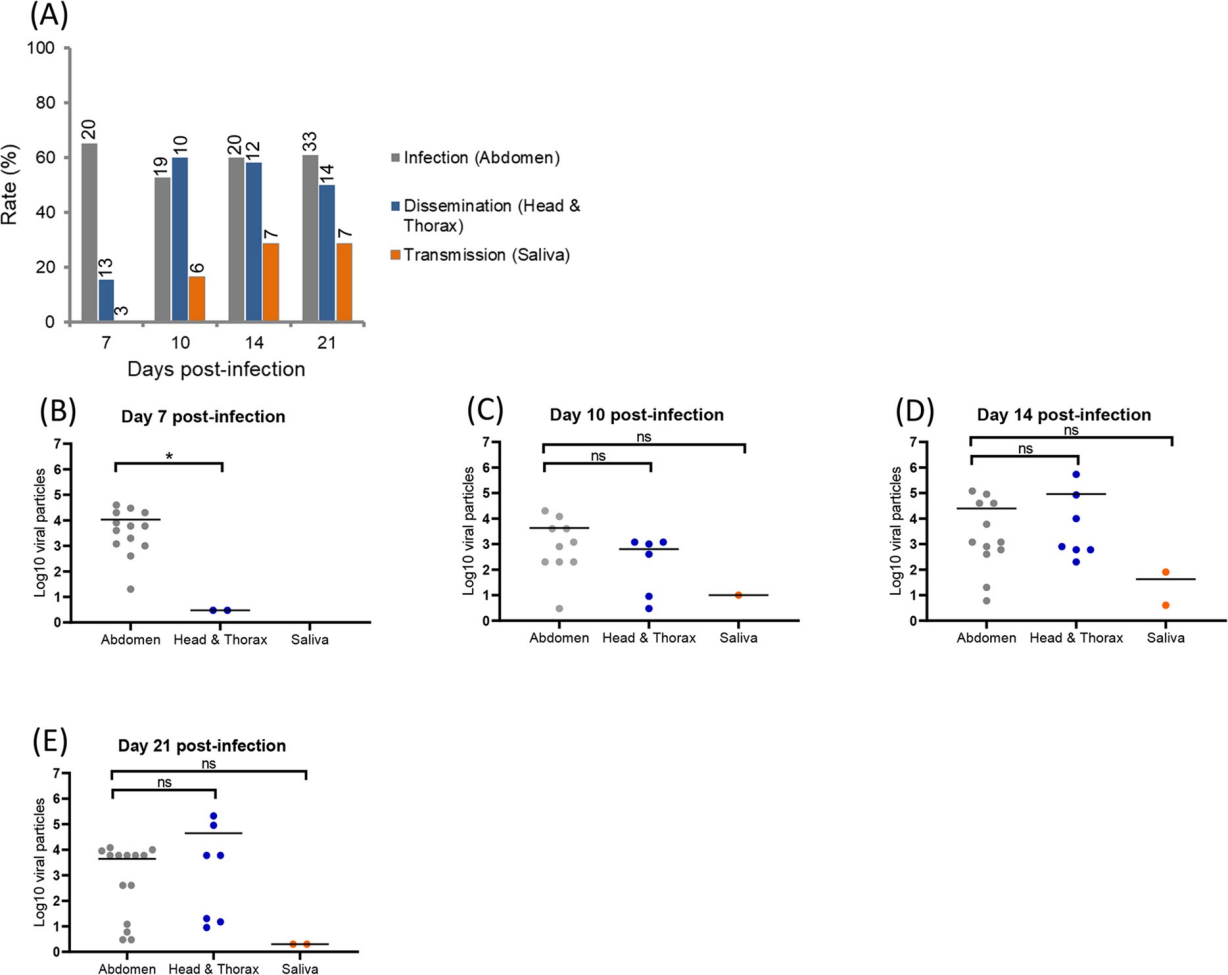
**ZIKV infection, dissemination, transmission (A) and viral loads in abdomen, head/thorax and saliva (B-F) in *Aedes albopictus* Tunisia at different days post-infection (3, 7, 10, 14 and 21).** Mosquitoes were infected with a blood meal at a titer of 10^7^ pfu/mL and processed as described in Fig 1. On top of the bars are the numbers of mosquitoes tested (A). Means are represented by horizontal bars (B-E). ns, non-significant, *p < 0.05.

When infected with ZIKV, IR ranged from 65% (7 dpi) to 60.9% (21 dpi), DR from 15.4% (7 dpi) to 50% (21 dpi), and TR from 16.7% (10 dpi) to 28.6% (21 dpi) (Fig 3A). Viral dissemination started from 7 dpi (DE of 10.5%) and transmission from 10 dpi (TE of 5.3%) (S1C Fig). When estimating viral loads in abdomen, head/thorax, and saliva at 7, 10, 14, and 21 dpi (Fig 3B–3F), they ranged from a mean of 10^4.0^ at 7 dpi to 10^3.6^ at 21 dpi in abdomen, from 10^0.5^ at 7 dpi to 10^4.6^ at 21 dpi in head/thorax, and from 10 at 10 dpi to 10^0.3^ at 21 dpi in saliva. Viral loads were similar in abdomen and head/thorax from 10 dpi (Mann-Whitney test: P > 0.05).

When estimating the time between the infectious blood meal and the detection of virus in mosquito saliva (namely the extrinsic incubation period, EIP), *Ae*. *albopictus* Tunisia mosquitoes were more efficient to transmit CHIKV, ZIKV and DENV, in descending order, with EIP of 3, 10 and 21 days respectively. Viral loads in abdomen and head/thorax were significantly higher for mosquitoes infected with CHIKV than with DENV and ZIKV (Mann-Whitney tests (2 by 2): P < 0.05, S2A and S2B Fig).

### A high viral load in the abdomen does not trigger necessarily viral dissemination and transmission

**Fig 4:**
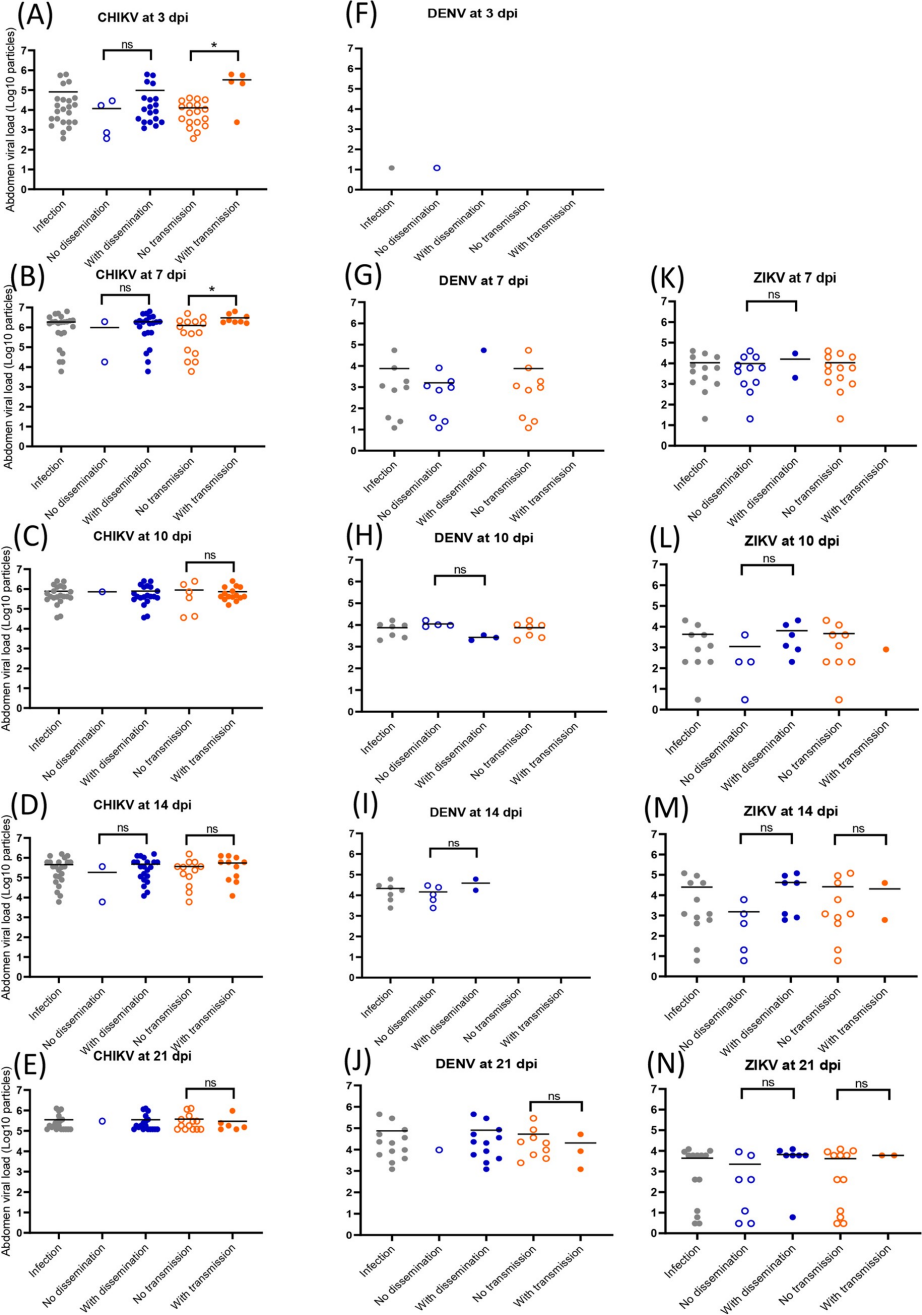
Viral loads in abdomen according to mosquito dissemination and transmission status: CHIKV (A-E), DENV (F-J) and ZIKV (K-N). Abdomen were homogenized and supernatants were titrated on cells. Means are represented by horizontal bars. ns, non-significant, *p < 0.05.

For CHIKV, DENV and ZIKV, no significant difference (Mann-Whitney test: P > 0.05) was detected suggesting no relation between viral load in abdomen and viral dissemination (Fig 4A–4N). When pooling all infected mosquitoes and building a ROC curve, the AUC (Area Under the Curve) corresponding to the surface below the ROC curve was 0.8305 showing that the viral load in abdomen discriminated moderately mosquitoes with and without viral dissemination (S3A Fig). The threshold that correctly classified the highest number of mosquitoes was 10^3.07^ viral particles in abdomen. Among mosquitoes with viral dissemination, 96% had more than 10^3.07^ viral particles in abdomen. However among mosquitoes without viral dissemination, 59% had more particles than this threshold. Of the 28 mosquitoes with viral particles below this threshold, 5 (17.9%) still disseminated. Nevertheless, a significant correlation (ρ = 0.72, P < 10^−4^) was detected between viral loads in abdomen and in head/thorax (S4 Fig).

To define if a high viral load detected in the abdomen can then be associated to viral transmission, viral loads in abdomen-infected mosquitoes were compared between individuals with transmission (i.e. virus detected in saliva) and without transmission (i.e. no virus detected in saliva). For CHIKV, mosquitoes able of viral transmission had a significantly higher number of viral particles in abdomen compared to mosquitoes without viral transmission (Mann-Whitney test: P < 0.05) at 3 dpi (10^5.5^ versus 10^4.1^; Fig 4A) and 7 dpi (10^6.5^ versus 10^6.1^; Fig 4B). It was not observed at later dpi (10 (Fig 4C), 14 (Fig 4D) and 21 (Fig 4E); Mann-Whitney test: P > 0.05). This pattern was not observed with DENV (Fig 4F–4J) and ZIKV (Fig 4K–4N). When building a ROC curve using all infected mosquitoes, the AUC was equal to 0.7111 showing that the viral load in abdomen weakly discriminated mosquitoes with and without transmission (S3B Fig). The threshold that correctly classified the highest number of mosquitoes was 10^5.6^ viral particles in abdomen. Among mosquitoes with viral transmission, 48% had more than 10^5.6^ viral particles in abdomen while among mosquitoes without viral transmission, 20% had viral particles above this threshold. The viral load in saliva was only moderately correlated with the viral load in abdomen (ρ = 0.33, P = 0.017) (S4B Fig).

### A high viral load in head/thorax does not trigger viral transmission

**Fig 5:**
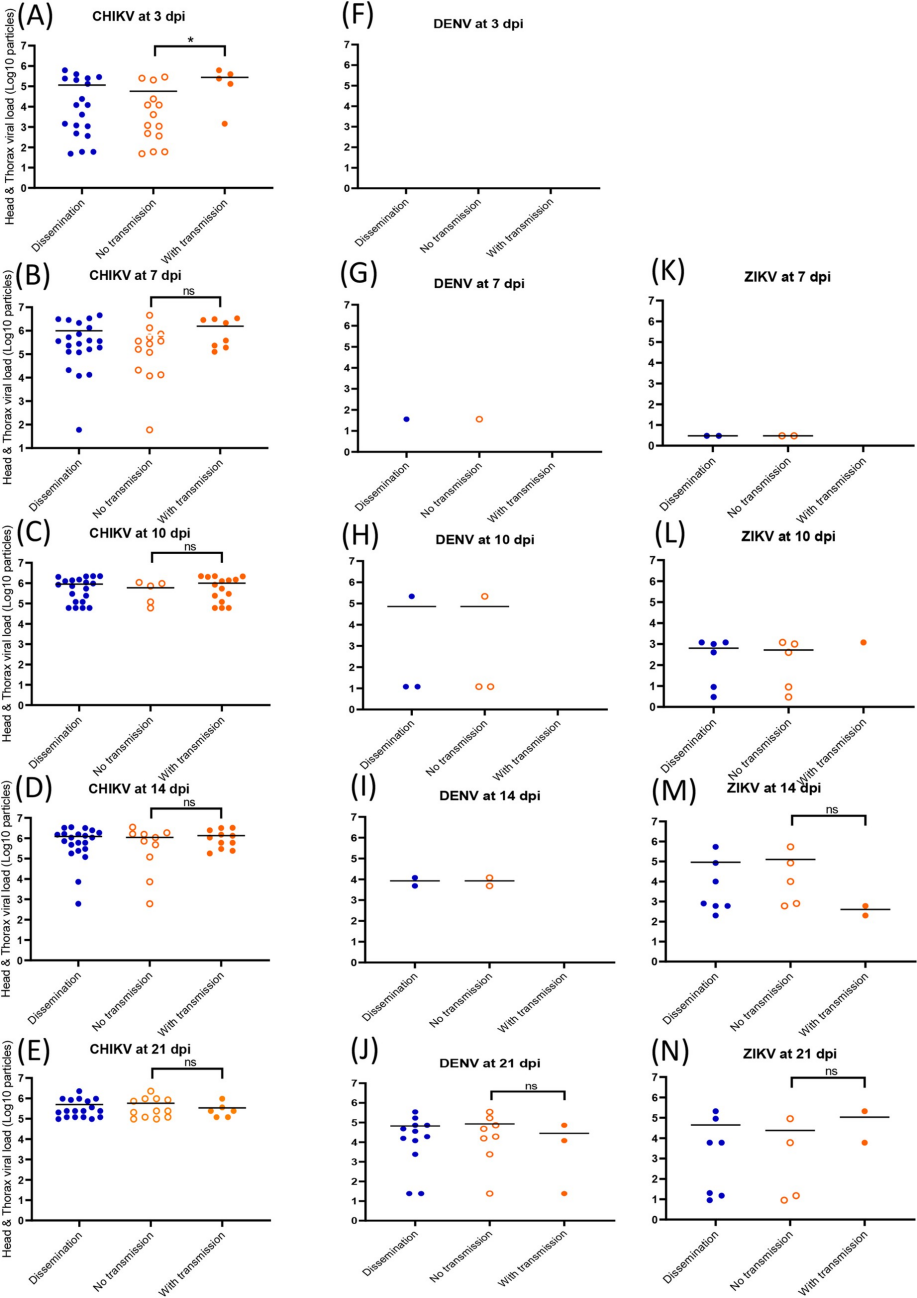
**Viral loads in head/thorax according to mosquito transmission status: CHIKV (A-E), DENV (F-J) and ZIKV (K-N).** Head/thorax were homogenized and supernatants were titrated on cells. Means are represented by horizontal bars. ns, non-significant, *p < 0.05, Mann-Whitney test.

For CHIKV (Fig 5A–5E), DENV (Fig 5F–5J) and ZIKV (Fig 5K–5N), there were no significant differences (Mann-Whitney test: P > 0.05) suggesting that there was no relation between viral load in head/thorax and transmission. In addition, a ROC curve was built using all mosquitoes with a disseminated infection (S3C Fig); the AUC value was 0.7102 showing that the viral load in head/thorax weakly discriminated mosquitoes with and without viral transmission. The threshold that correctly classified the highest number of mosquitoes was 10^5.1^ viral particles in head/thorax. Among mosquitoes with viral transmission, 74% had more than 10^5.1^ viral particles in head/thorax while among mosquitoes without viral transmission, 39% had viral particles above this threshold. The correlation between viral load in head/thorax and in saliva was not significant (ρ = 0.21, P = 0.14).

### *Aedes albopictus* mosquitoes from Tunisia are as competent as *Ae*. *albopictus* La Réunion

**Fig 6:**
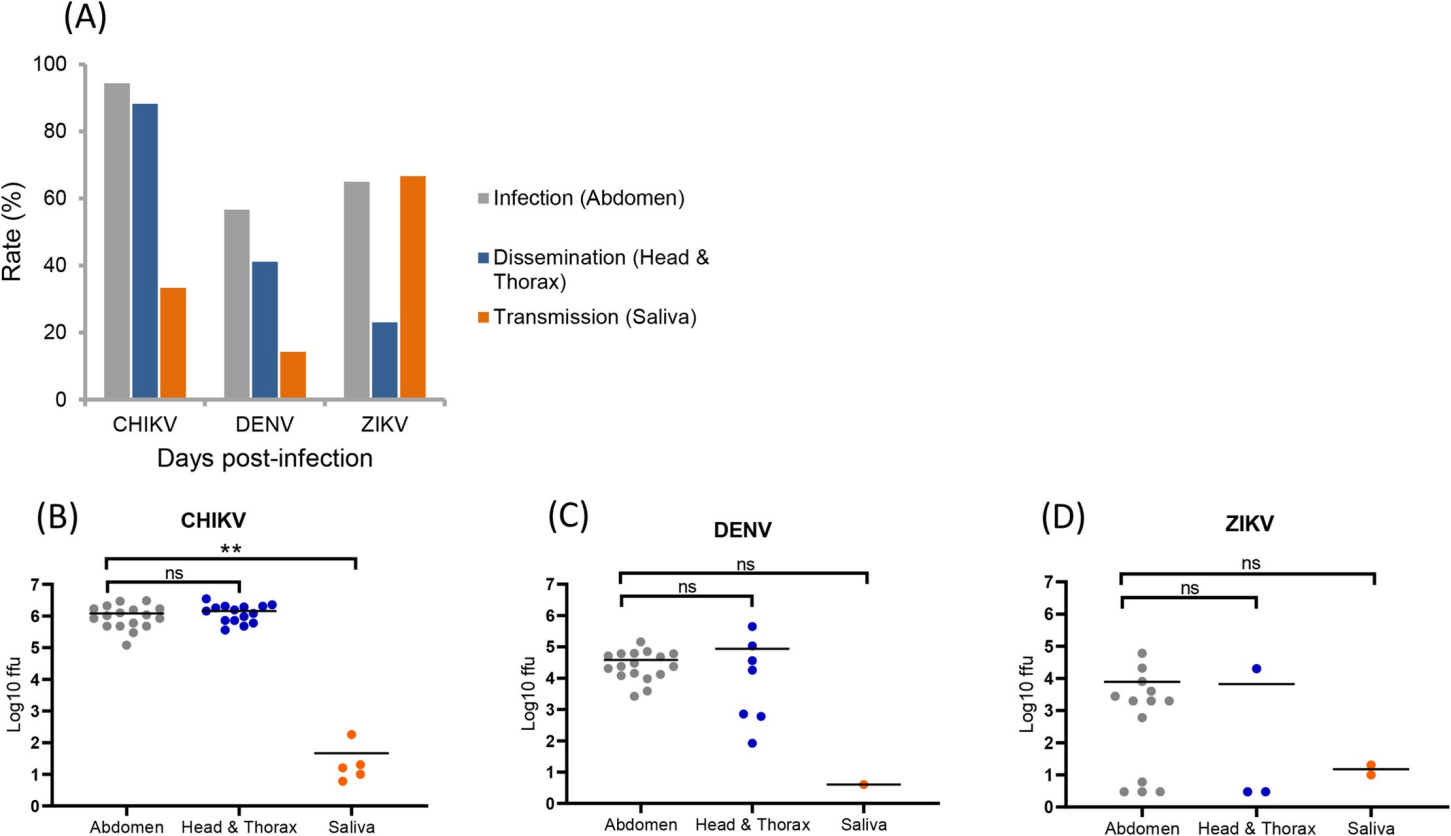
**Infection, dissemination, transmission (A) and viral loads in abdomen, head/thorax and saliva (B-D) in *Aedes albopictus* La Réunion, 14 days after infection with CHIKV, DENV and ZIKV.** Mosquitoes were infected with a blood meal at a titer of 10^7^ ffu/mL and at 14 days post-infection, mosquitoes were processed to estimate the viral load in abdomen, head/thorax and saliva by titration on cells. Means are represented by horizontal bars. ns, non-significant, **p ≤ 0.01, Kruskal-Wallis test.

Fig 6A shows IR, DR, and TR at 14 dpi for *Ae*. *albopictus* La Réunion. IRs were 94.4% for CHIKV, 65% for ZIKV and 56.7% for DENV and among abdomen-infected mosquitoes, CHIKV induced the highest DR in mosquitoes (88.2%) while lower DRs were measured for DENV (41.2%) and ZIKV (23.1%). Among mosquitoes with disseminated infection, ZIKV produced the highest TR (66.7%) followed by CHIKV (33.3%) and DENV (14.3%). When considering more globally viral dissemination and transmission among tested mosquitoes, values of DE and TE were respectively for CHIKV (88.3%, 27.8%), DENV (23.3%, 3.3%), and ZIKV (15%, 10%) showing that CHIKV was more efficient to disseminate and be transmitted (S5 Fig). When estimating the number of viral particles in abdomen, head/thorax, and saliva at 14 dpi (Fig 6B–6D), no significant differences in viral loads were found between abdomen (CHIKV: a mean of 10^5.9^, DENV: 10^4.4^, ZIKV: 10^2.7^) and head/thorax (CHIKV: 10^6.1^, DENV: 10^3.9^, ZIKV: 10^1.7^) (Mann-Whitney test: P > 0.05). The viral loads in saliva were much lower: 10^1.3^ for CHIKV, 10^0.6^ for DENV and 10^1.1^ for ZIKV.

**Fig 7:**
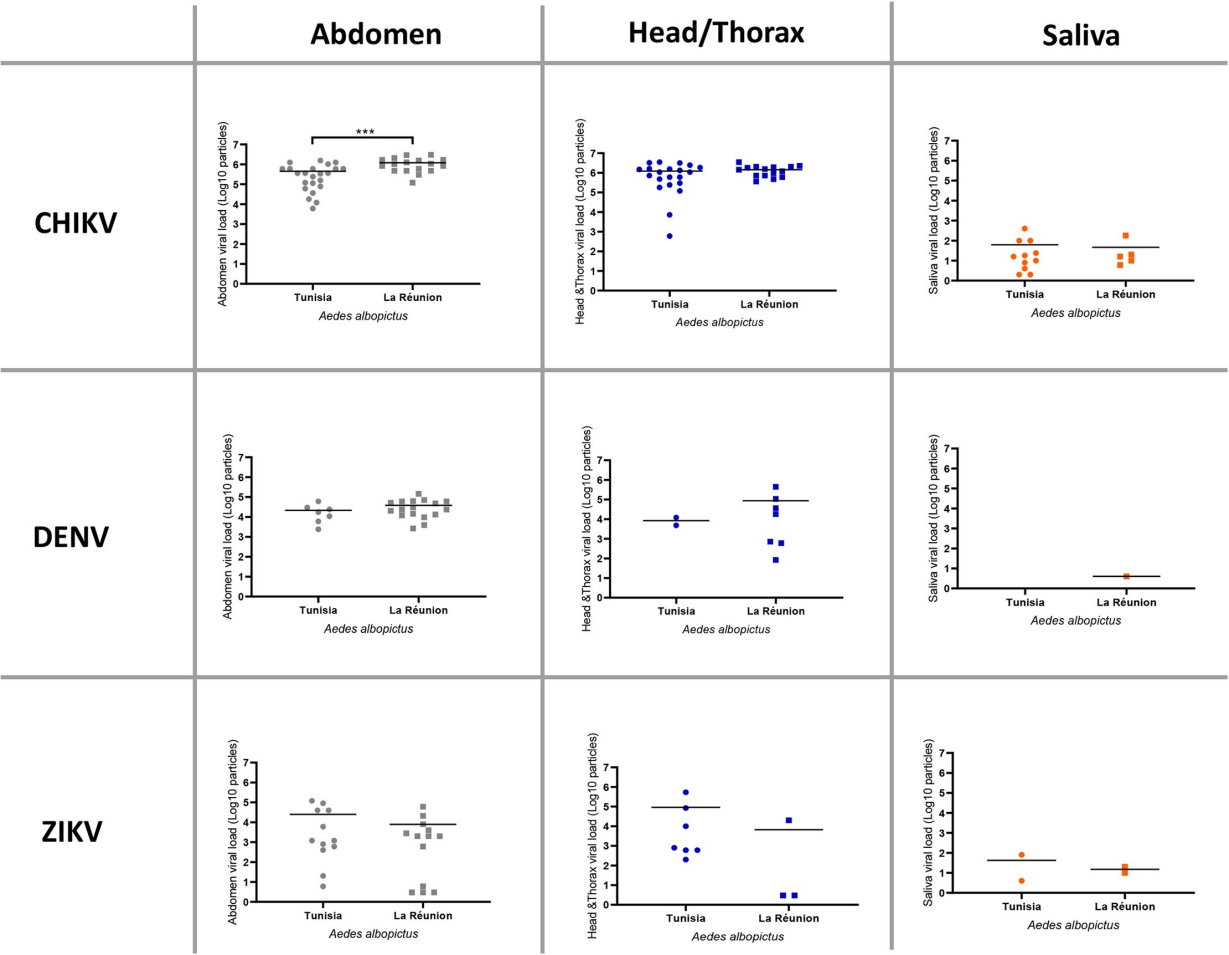
Comparison between *Aedes albopictus* Tunisia and *Aedes albopictus* La Réunion for viral loads in abdomen, head/thorax and saliva 14 days after infection with CHIKV, DENV, and ZIKV. Means are represented by horizontal bars. ***p ≤ 0.001, Mann-Whitney test.

When comparing with data obtained at 14 dpi for *Ae*. *albopictus* Tunisia, all parameters measured did not show any significant differences: rates/efficiencies (IR, DE, DR, TE, TR) (Fisher’s exact test: P > 0.05) and viral loads (in abdomen, head/thorax, saliva) (Mann-Whitney test: P > 0.05). There were two exceptions, IR for DENV (p = 0.004) and viral load in abdomen for CHIKV (p = 0.001) (Fig 7).

## Discussion

*Aedes albopictus* mosquitoes from Tunisia were more susceptible to CHIKV than to ZIKV and DENV. While CHIKV particles can be detected in mosquito saliva from 3 dpi, ZIKV and DENV require a longer period of incubation for transmission to occur, respectively 10 and 21 days. *Ae*. *albopictus* mosquitoes from Tunisia were as competent as *Ae*. *albopictus* La Réunion, a colony initially collected in an epidemic region for CHIKV and DENV.

*Aedes albopictus* became the main vector of CHIKV following the selection of a single mutation (an amino-acid change at the position 226 in the E1 glycoprotein (E1-A226V); [19, 21, 22]). In Europe where *Ae*. *albopictus* is established in 20 countries [47], this mosquito has been responsible since 2007, for local transmission of CHIKV, DENV [12] and ZIKV [15]. A prediction risk map based on vector competence data and vector distribution stresses the high risk of CHIKV epidemics in Europe associated with *Ae*. *albopictus*, more than with DENV and ZIKV [48]. Collectively, European *Ae*. *albopictus* mosquitoes require at least 2–3 days for viral transmission to occur after the infectious blood meal [49] as did mosquitoes from La Réunion with a maximum of 10^3.3^ infectious viral particles at day 6 post-infection [45]. When changing parameters such as the incubation temperature after the infectious blood meal or the CHIKV genotype used for mosquito infections [50], the outcome of infection became significantly different suggesting that the CHIKV transmission potential by *Ae*. *albopictus* depends on the three-way combination of mosquito population, virus strain and temperature [51]. Indeed, when infected with the same CHIKV strain (06.21), transmission was higher for *Ae*. *albopictus* Tunisia than for *Ae*. *albopictus* from Morocco [12] but much lower when compared with mosquitoes from Cameroon [52] or Brazil [53]. At 3 and 7 dpi, it has been demonstrated that a high CHIKV load in the midgut triggers viral transmission and not dissemination. The midgut seems to play a secondary role as a barrier compared to the salivary glands where active replication leads to a high transmission rate [50]. In patients, CHIKV infections induce a high level of viremia ranging from 10^4^−10^8^ RNA copies/mL [54, 55] and including the viral titer of the infectious blood meal used.

ZIKV produced the first local cases in Europe (in France) in August 2019 [15]. This was unexpected as during the last Zika pandemic when hundreds of imported cases were reported in France in 2016 [56], no autochthonous human cases were reported in France and more globally, in Europe. Three years later, in October 2019, while few active outbreaks of Zika were observed in the world, three autochthonous Zika cases were reported in the Var (France) [15]. *Aedes albopictus* mosquitoes from Europe require at least 7–14 days to excrete viral particles in saliva [29, 30, 57, 58], which is consistent with our 10 days. *Aedes albopictus* Tunisia required less time to transmit ZIKV compared to *Ae*. *albopictus* Morocco which needed 21 days [12]. Moreover, low relation was detected between viral loads in the three compartments, abdomen, head/thorax and saliva meaning that a threshold of viral loads was not the only condition required to trigger viral dissemination and transmission, at least in the pairing *Ae*. *albopictus* Tunisia and Asian genotype of ZIKV. When infected with the Asian genotype of ZIKV, *Ae*. *albopictus* mosquitoes from France developed viral loads insufficiently high in midgut and salivary glands to trigger dissemination and then, transmission [57]. It has been shown that *Ae*. *albopictus* mosquitoes from France transmit 10 to 20 times better the African genotype of ZIKV than the Asian genotype [57] stressing the specific outcome of each pairing mosquito population and virus strain [59]. Interestingly, *Ae*. *albopictus* from Cameroon transmit very efficiently the West African genotype of ZIKV [60], a critical point that may contribute to increase opportunities for the West African ZIKV to be exported from the country *via* viremic travelers as it happened with CHIKV [61].

Approximately 242 dengue infections were imported to Europe by returning travelers from 2012 to 2014 [62]. Up to 36% of dengue-infected travelers became symptomatic after their return and 58% of the patients with acute infections were viremic [62]. The number of imported dengue cases has been increasing in Europe with 2033 cases in 2018 [63]. This poses a risk for autochthonous transmission of dengue in European regions where *Ae*. *albopictus* mosquitoes are present. The fear became reality in 2010 when local dengue cases were reported in France [64], Croatia [65], and Spain [66]. Further autochthonous dengue cases were reported in France in 2013 [67], 2015 [68], and 2018 [69]. Moreover, DENV was detected in a pool of *Ae*. *albopictus* at the vicinity of a viremic traveler in Spain [70]. *Aedes albopictus* mosquitoes from Tunisia were weakly competent to DENV necessitating 21 days to excrete viral particles in saliva, which exceeded the 14 days necessary for *Ae*. *albopictus* Morocco to transmit DENV [12]. This mosquito was also less competent to DENV than mosquitoes from Cameroon and Gabon [71, 72].

To conclude, the recent establishment of *Ae*. *albopictus* in Tunisia poses the threat of emergence of CHIKV and to a lesser extent, of ZIKV and DENV considering the vector competence data obtained. Introductions of viremic travelers from endemic countries for these viruses *via* migrant workers or tourists may initiate local transmission when environmental conditions are favorable to sustain high vector densities at proximity of a high proportion of immunologically naive humans. Therefore, control of vectors and surveillance of febrile travelers should be reinforced during the season of high attendance of Tunisia by tourists.

## Supporting information

S1 Fig Dissemination and transmission efficiencies at different days (3, 7, 10, 14 and 21) after infection of *Aedes albopictus* Tunisia with CHIKV (A), DENV (B) and ZIKV (C). Dissemination efficiency refers to the proportion of mosquitoes with virus detected in head/thorax among the total number of mosquitoes examined, and transmission efficiency to the proportion of mosquitoes with virus detected in saliva among all mosquitoes examined.
(PDF)

S2 Fig Viral loads in abdomen (A) and head/thorax (B) after infection of *Ae*. *albopictus* Tunisia with CHIKV, DENV, and ZIKV. Mosquitoes were infected with a blood meal at a titer of 10^7^ ffu/mL and were processed to estimate the viral load in abdomen and head/thorax by titration on cells. Means are represented by horizontal bars.
(PDF)

S3 Fig ROC curves to identify mosquitoes capable of viral dissemination (A) according to viral load in abdomen, and viral transmission according to viral load in abdomen (B) and head/thorax (C).
(PDF)

S4 Fig Correlation between viral load in abdomen and viral load in head/thorax (A), and between viral load in abdomen and viral load in saliva (B).
(PDF)

S5 Fig Dissemination and transmission efficiencies, 14 days after infection of *Aedes albopictus* La Réunion with CHIKV, DENV and ZIKV. (PDF)
