# Supplementary figures and images for: The recently introduced Aedes albopictus in Tunisia has the potential to transmit chikungunya, dengue and Zika viruses
Source: PLoS Negl Trop Dis. 2020 Oct 2;14(10):e0008475. doi: 10.1371/journal.pntd.0008475 (PMC7556531; doi:10.1371/journal.pntd.0008475)

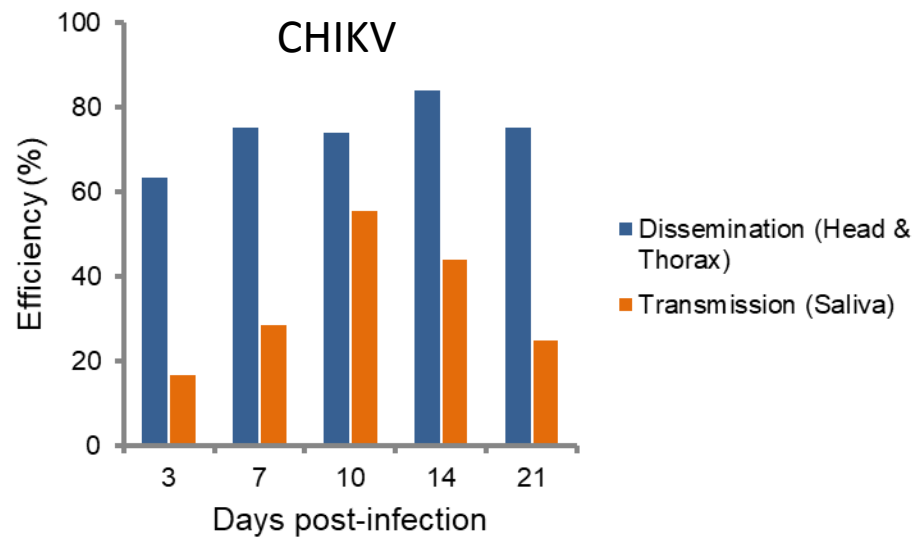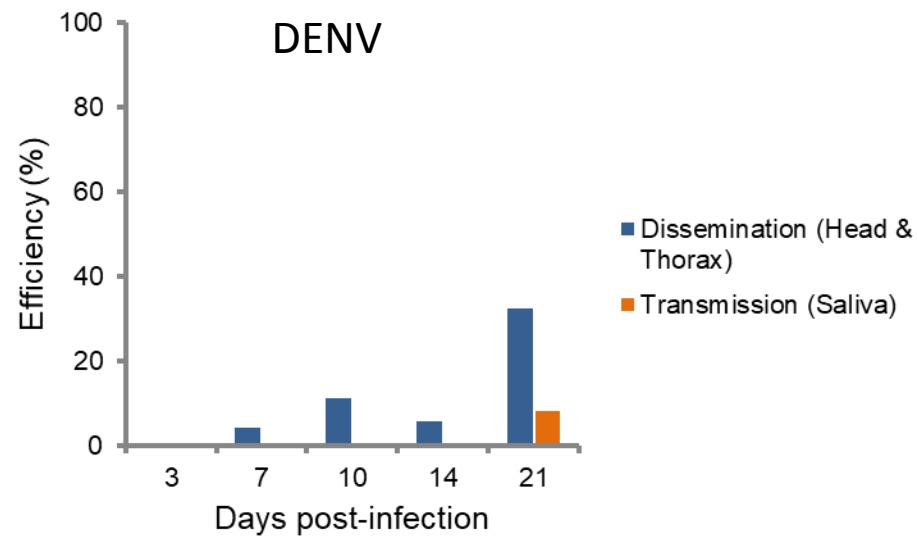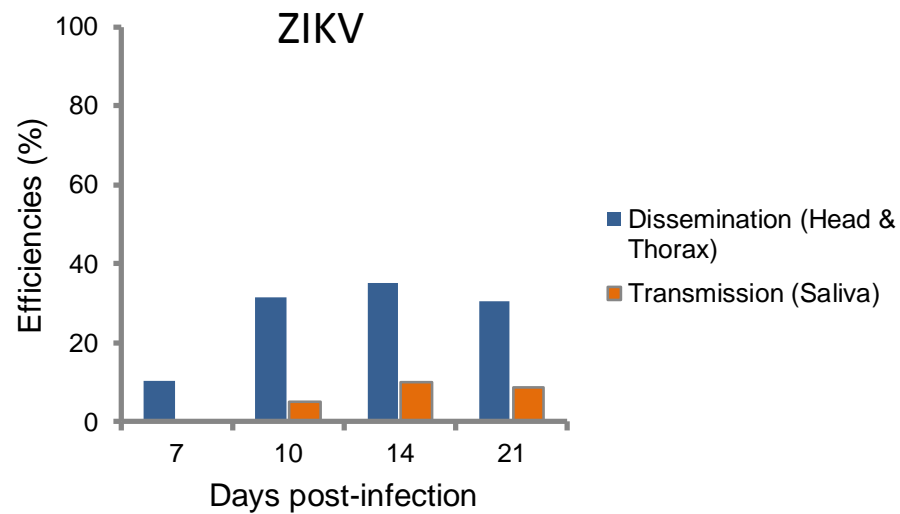

Supplement: S1 Fig — Dissemination and transmission efficiencies at different days (3, 7, 10, 14 and 21) after infection of Aedes albopictus Tunisia with CHIKV (A), DENV (B) and ZIKV (C). Dissemination efficiency refers to the proportion of mosquitoes with virus detected in head/thorax among the total number of mosquitoes examined, and transmission efficiency to the proportion of mosquitoes with virus detected in saliva among all mosquitoes examined. (PDF) [file pntd.0008475.s001.pdf]

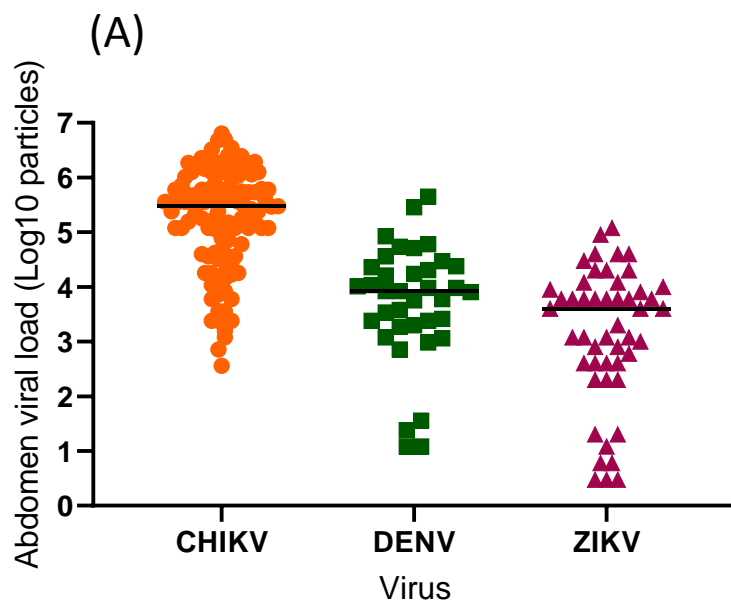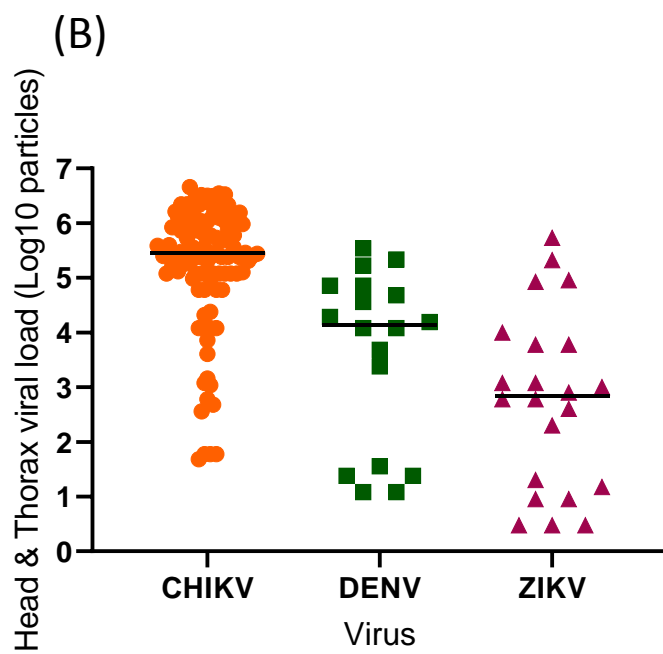

Supplement: S2 Fig — Viral loads in abdomen (A) and head/thorax (B) after infection of Ae. albopictus Tunisia with CHIKV, DENV, and ZIKV. Mosquitoes were infected with a blood meal at a titer of 107 ffu/mL and were processed to estimate the viral load in abdomen and head/thorax by titration on cells. Means are represented by horizontal bars. (PDF) [file pntd.0008475.s002.pdf]

(A)

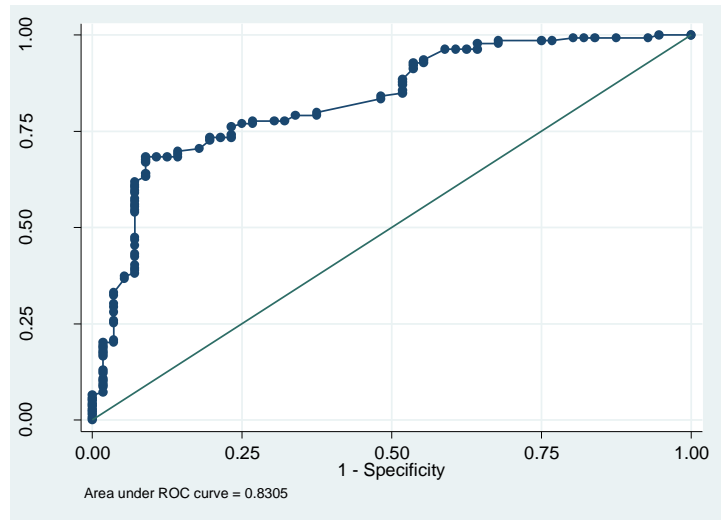

(B)

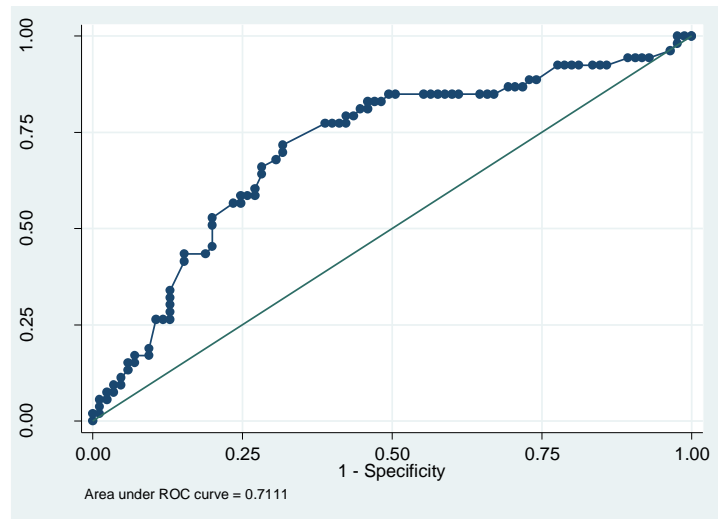

(C)

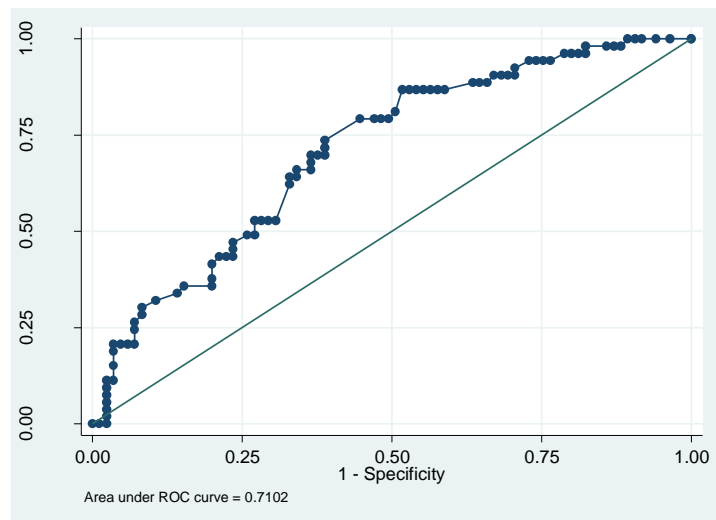

Supplement: S3 Fig — ROC curves to identify mosquitoes capable of viral dissemination (A) according to viral load in abdomen, and viral transmission according to viral load in abdomen (B) and head/thorax (C). (PDF) [file pntd.0008475.s003.pdf]

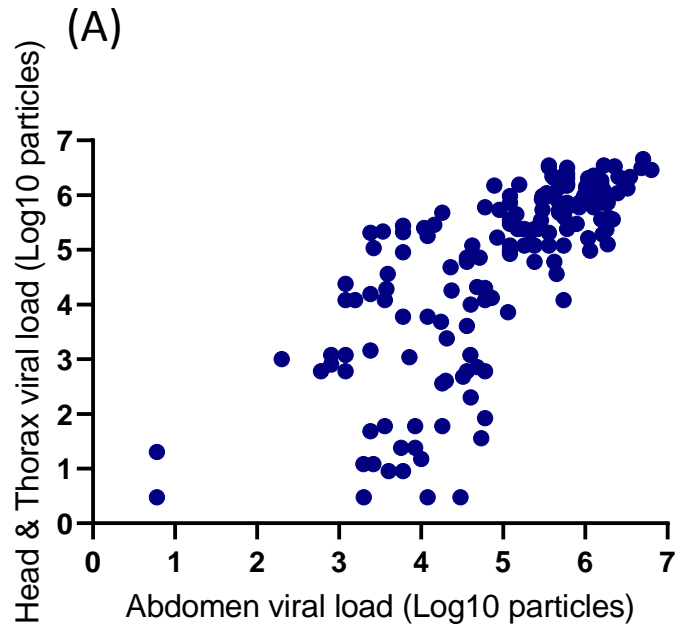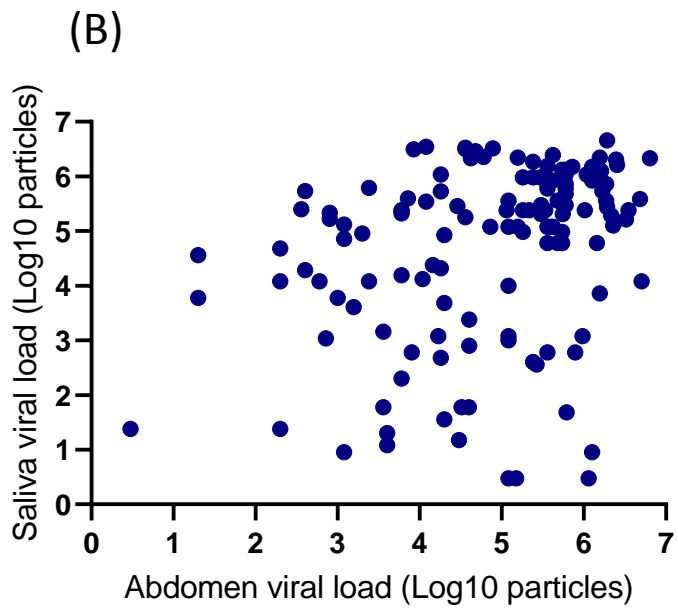

Supplement: S4 Fig — Correlation between viral load in abdomen and viral load in head/thorax (A), and between viral load in abdomen and viral load in saliva (B). (PDF) [file pntd.0008475.s004.pdf]

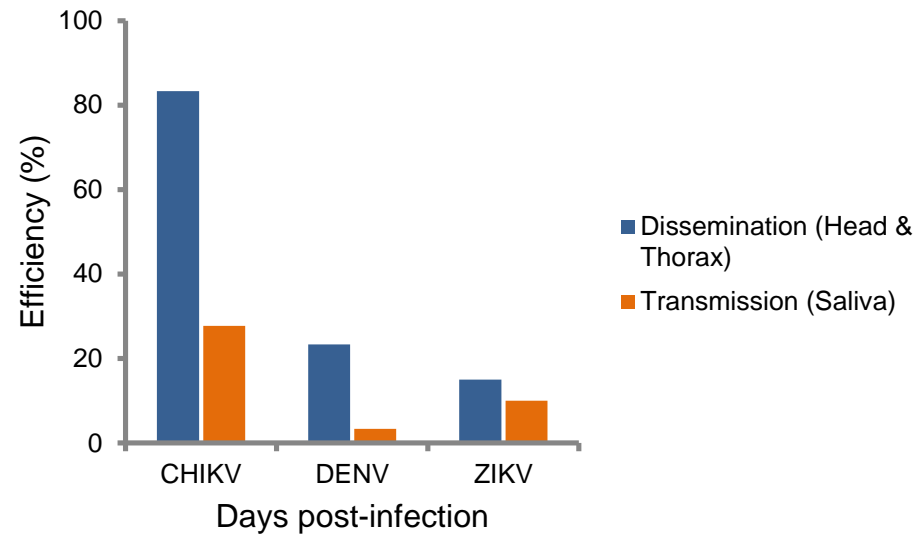

Supplement: S5 Fig — (PDF) [file pntd.0008475.s005.pdf]
